# Assessment of Two Novel Live-Attenuated Vaccine Candidates for Herpes Simplex Virus 2 (HSV-2) in Guinea Pigs

**DOI:** 10.3390/vaccines9030258

**Published:** 2021-03-13

**Authors:** Jonathan D. Joyce, Anant K. Patel, Brandie Murphy, Daniel J.J. Carr, Edward Gershburg, Andrea S. Bertke

**Affiliations:** 1 Translational Biology, Medicine and Health, Virginia Polytechnic Institute & State University, Blacksburg, VA 24061, USA; jjoyce84@vt.edu; 2 Department of Population Health Sciences, Virginia Maryland College of Veterinary Medicine, Virginia Polytechnic Institute & State University, Blacksburg, VA 24061, USA; anantpatel96@vt.edu; 3 Rational Vaccines, Inc., Cambridge, MA 02139, USA; brandie.murphy@rationalvaccines.com (B.M.); ed.gershburg@rationalvaccines.com (E.G.); 4 Departments of Microbiology, Immunology, and Ophthalmology, University of Oklahoma Health Sciences Center, Oklahoma City, OK 73034, USA; dan-carr@ouhsc.edu

**Keywords:** herpes simplex virus, HSV-2, vaccine, latency, dorsal root ganglia, guinea pig, immunofluorescence, fluorescent in situ hybridization, explant reactivation, RT-qPCR, qPCR

## Abstract

Treatment to ameliorate the symptoms of infection with herpes simplex virus 2 (HSV-2) and to suppress reactivation has been available for decades. However, a safe and effective preventative or therapeutic vaccine has eluded development. Two novel live-attenuated HSV-2 vaccine candidates (RVx201 and RVx202) have been tested preclinically for safety. Hartley guinea pigs were inoculated vaginally (*n* = 3) or intradermally (*n* = 16) with either vaccine candidate (2 × 10^7^ PFU) and observed for disease for 28 days. All animals survived to study end without developing HSV-2-associated disease. Neither vaccine candidate established latency in dorsal root or sacral sympathetic ganglia, as determined by viral DNA quantification, LAT expression, or explant reactivation. Infectious virus was shed in vaginal secretions for three days following vaginal inoculation with RVx202, but not RVx201, although active or latent HSV-2 was not detected at study end. In contrast, guinea pigs inoculated with wild-type HSV-2 MS (2 × 10^5^ PFU) vaginally (*n* = 5) or intradermally (*n* = 16) developed acute disease, neurological signs, shed virus in vaginal secretions, experienced periodic recurrences throughout the study period, and had latent HSV-2 in their dorsal root and sacral sympathetic ganglia at study end. Both vaccine candidates generated neutralizing antibody. Taken together, these findings suggest that these novel vaccine candidates are safe in guinea pigs and should be tested for efficacy as preventative and/or therapeutic anti-HSV-2 vaccines.

## 1. Introduction

HSV-2 remains the leading cause of genital herpes and is associated with 60% of genital ulcers in the United States, Asia, Africa, and parts of Europe [1]. The epidemiology of genital herpes is changing, however, as first-time genital herpes infections in industrialized, high income countries are increasingly associated with HSV-1, especially among individuals under 25 years of age, college students, females, and men who have sex with men. This is likely driven by reduced prevalence of early childhood HSV-1 infection, leaving adolescents and young-adults susceptible to genital infection when they become sexually active [1,2,3,4]. Genital herpes caused by HSV-2, however, has a markedly different clinical progression than that caused by HSV-1, characterized by greater frequencies of clinical recurrences and episodes of asymptomatic viral shedding [1,3,5,6]. HSV-2 infects an estimated 13% of the global population and the World Health Organization estimates that nearly half a billion people 15–49 years of age are infected with HSV-2 [7]. The infection disproportionately impacts women worldwide [8]. In the United States, at least one in six Americans aged 14–49 years are currently infected, disproportionately impacting women (15.9% prevalence in women compared to 8.9% in men) and African Americans [9].

Infection with HSV-2 is lifelong, as the virus establishes latency in sensory neurons of the lumbosacral dorsal root ganglia (DRG) and autonomic neurons innervating the genitourinary system [10]. Latent infections can reactivate periodically due to any number of internal or external stimuli, including stress, illness, or menstruation, resulting in painful lesions at or near the site of infection. During reactivation, approximately 20% of patients also develop non-genital involvement, including lesions on the lumbosacral back, buttocks, and legs [11]. Primary infections and recurrences can also result in keratoconjunctivitis, aseptic meningitis, encephalitis, disseminated infection (including the visceral organs), or secondary bacterial infections of ulcerated skin, some of which can be fatal if not properly diagnosed and managed [12,13,14,15,16,17]. In immunocompromised patients, recurrences can be even more severe, sometimes requiring surgical intervention to remove expanding lesions [18]. Importantly, infection with HSV-2 has been linked to increased risk of infection with HIV [19,20,21,22]. Also of importance, HSV-2 can be transmitted from an infected mother to neonates during or after delivery causing neonatal herpes, which complicates 1:2000–13,000 live births in the United States annually. Systemic neonatal herpes is fatal in up to 60% of cases and can produce severely debilitating sequelae in survivors, including developmental and cognitive delays, seizure disorders, and blindness [23,24].

Treatment options to ameliorate the symptoms of HSV-2 infection rely on acyclovir and its derivatives. These drugs are typically taken daily by mouth with dosing determined by the nature of the infection (primary vs. recurrent) and the goal of treatment (acute or prophylactic management). These antivirals can limit the frequency, severity, and duration of recurrences; however, they are not curative and treatment efficacy is dependent on the timing of drug delivery during the course of clinical disease [25,26,27]. As with any viral infection, the selective pressure applied by the use of antiviral drugs can drive resistance in the viral population, especially in the immunocompromised (up to 5% in such cases) [26,28,29].

Safe sex practices and antiviral therapies are the cornerstone of HSV-2 prevention; however, prevention of infection is complicated by the fact that transmission can occur when the infected person is shedding virus but has no outward clinical manifestations of disease. Viral shedding is accompanied by clinical disease only ≈20% of the time, allowing for the asymptomatic transmission of infection, which presents a challenge to infection control and prevention measures [30,31,32,33]. It is clear that preventative measures are needed beyond what is currently available.

To that end, a preventative or therapeutic HSV-2 vaccine has long been a goal of herpes prevention. Several vaccine candidates using various platforms have been tested in clinical trials in humans but have failed to prevent HSV-2 infection in any meaningful way. Most vaccine candidates studied have been subunit vaccines or replication deficient viruses, which do not present the full range of viral antigens and are therefore unable to elicit a truly robust protective immune response. With that in mind, a live-attenuated HSV-2 vaccine is an attractive alternative to previous vaccine candidates. However, they are infrequently investigated due to the perceived fear of reversion of the vaccine to wild type HSV-2, as well as interference of virus-specific immune evasion responses [34,35,36,37,38]. The success of a live-attenuated vaccine for prevention of varicella zoster virus, an alphaherpesvirus that establishes latency in sensory and autonomic ganglia similarly to HSV, sets a precedent for the use of a live-attenuated HSV-2 vaccine.

In response to the need for a preventative and/or therapeutic HSV-2 vaccine and the lack of currently viable live-attenuated anti-HSV-2 based vaccine candidates, we report the pre-clinical safety validation of two novel live-attenuated HSV-2 vaccine candidates. The vaccine candidates were found to be safe in a guinea pig model of infection. Further development of these vaccine candidates represents a response to the public health need for a vaccine to therapeutically treat recurrences, or potentially prevent, one of the most stigmatized sexually transmitted infections.

## 2. Materials and Methods

### 2.1. Cells & Viruses

Stocks of RVx201, RVx202, wild type (WT) HSV-2 strain MS, and Vero cell lysates were provided by Rational Vaccines Inc. (Cambridge, MA). The WT HSV-2 strain MS was isolated in 1961 from the midbrain of a 50-year-old Icelandic female with a protracted history of multiple sclerosis [39]. RVx201 and RVx202 were generated as previously described, by targeted mutations throughout the coding region of infected cell protein 0 (ICP0) using HSV-2 MS as a backbone [40]. WT HSV-2 stocks were generated using Vero cells from American Type Culture Collection (ATCC) while RVx201 and RVx202 stocks were generated using U2OS cells (ATCC). Cells were maintained in Dulbecco’s Modified Eagle’s medium (DMEM) containing 5% fetal bovine serum (FBS) and 100 U/mL penicillin G and streptomycin. Standard plaque assays were used to verify viral titers using Vero cells for WT HSV-2 and ICP0-complementing cells for RVx201 and RVx202 (Neal Deluca, University of Pittsburgh [41]). All cell lines cells were maintained following standard cell culture techniques for use in plaque assays.

### 2.2. Guinea Pig Inoculation, Clinical Assessment and Tissue Collection

3–4-week-old female Hartley guinea pigs (Charles River Laboratories, Wilmington MA) were inoculated under anesthesia (ketamine/xylazine) vaginally (VAG) by pipette or intradermally (ID) by tuberculin syringe (*n* = 16 each group) with RVx201 (2 × 10^7^ PFU/mL, *n* = 16 ID, *n* = 3 VAG)), RVx202 (2 × 10^7^ PFU/mL, *n* = 16 ID, *n* = 3 VAG)), WT HSV-2 MS (2 × 10^5^ PFU/mL, *n* = 16 ID, *n* = 5 VAG)); or were inoculated ID with an equivalent volume of Vero cell lysate (*n* = 3), or were mock infected with PBS (*n* = 3). Guinea pigs were assessed daily for weight gain/loss and signs of distress and/or disease for 28 days post inoculation (dpi). Genital disease was assessed on a well-established 4-point severity scale, with 0 = no disease, 1 = redness and/or swelling, 2 = 1–2 lesions, 3 = 3–5 lesions, 4 = 6 or more lesions or coalescence of lesions, and recurrences were defined as presence of vesicular lesions on the first day of appearance. Clinical disease following intradermal inoculation was assessed on a similar 4-point severity scale, assessing for presence of clinical symptoms on the inner thigh at or near the site of inoculation. Neurological signs were assessed as presence of urinary retention and hindlimb weakness or paralysis each day. Guinea pigs were vaginally swabbed daily for quantification of viral shedding in vaginal secretions using FLOQSwabs (Copan Diagnostics, Murrieta, CA, USA) moistened with Dulbecco’s Modified Eagle Medium (DMEM, Thermo Fisher, Waltham MA, USA). Swabs in DMEM were stored at −80 °C for assessment via plaque assay. At study end, following euthanasia, sensory and autonomic ganglia including lumbosacral dorsal root ganglia (LS-DRG) sacral sympathetic ganglia (SSG), trigeminal ganglia (TG), superior cervical ganglia (SCG), brain and injection site skin were collected to assess for the presence of HSV-2 by qPCR and immunofluorescence (IF), expression of LAT by qRT-PCR and fluorescent in situ hybridization (FISH), and the ability to reactivate by explant reactivation. Blood was collected by cardiac puncture into serum separator tubes and centrifuged for 10 min at 3000 rpm. Sera and tissues, including brain, liver, kidney, and injection site skin, were collected in 4% PFA and subjected to immunological and histological analyses.

### 2.3. Assessment Of HSV-2 Shedding in Vaginal Secretions

Vero or U2-OS cells (ATCC) were used for standard plaque assay of daily guinea pig vaginal swabs for the assessment of viral shedding in vaginal secretions. Plaque assays were completed in duplicate in 24-well plates for each vaginal swab sample.

### 2.4. Explant Reactivation

Ganglia including LS-DRG, SSG, TG, and SCG from five ID inoculated and one VAG inoculated guinea pig(s) as well as ganglia from one guinea pig inoculated with Vero cell lysates and one guinea pig inoculated with PBS were reduced to single cell suspensions following enzymatic digestion and trituration, then plated onto Matrigel-coated culture plates and incubated at 37 °C with 5% CO_2_ for seven days as previously described [42]. Latent viral reactivation in the brain from the above animals was assessed by plating a homogenate of the left hemisphere, which was cultured as described above. An aliquot of media was collected from half of the plates on alternating days for seven days and assessed for the presence of infectious HSV-2 via plaque assay as described above using U2-OS or Vero cells.

### 2.5. Thymidine kinase (TK)-specific qPCR and LAT-specific RT-qPCR

Ganglia including LS-DRG, SSG, TG, and SCG from five guinea pigs inoculated ID and one guinea pig inoculated VAG (two for WT HSV-2 MS) from each group as well from one guinea pig inoculated with Vero cell lysate and one guinea pig inoculated with PBS were collected in RLT buffer in bead tubes and homogenized using a BeadBug3 (Benchmark Scientific, Sayreville, NJ, USA). DNA and RNA were extracted using an AllPrep DNA/RNA Mini Kit (Qiagen, Germantown, MD, USA) following the manufacturer’s protocol. For use in RT-qPCR specific for LAT, complementary DNA (cDNA) was produced in 20 µL reactions from RNA using the iScript cDNA Synthesis Kit (Bio-Rad, Hercules, CA, USA) following the manufacturer’s protocol. For qPCR specific for TK2, iTaq Universal Probe Supermix (Bio-Rad, Hercules CA, USA) was used in 10 μL reactions and run on a Viia 7 real-time PCR system (Applied Biosystems, Foster City CA, USA). TK-specific qPCR was run using the fast setting, 95 °C for 20 s (one cycle); 95 °C for 1 s and 60 °C for 20 s (40 cycles). LAT-specific qPCR was run using the standard setting, 50 °C for 2 min and 95 °C for 10 min (one cycle); 95 °C for 15 s and 60 °C for 60 s (40 cycles). Results were reported as HSV-2 RNA copies/200 ng total RNA or DNA based on sample ct values as they related to ct values of genomic standards of known concentration.

### 2.6. Immunofluorescence (IF) and Fluorescent in Situ Hybridization (FISH)

*Tissue preparation:* Ganglia, including bilateral LS-DRG, SSG, TG and SCG were collected from three guinea pigs inoculated ID from each virus-infected group (RVx201, RVx202, HSV-2 MS), one guinea pig inoculated vaginally from each of these groups, one guinea pig mock-infected with Vero cell lysate, and one mock-infected with PBS. Tissues were fixed in 4% PFA overnight, transferred into 30% sucrose overnight, embedded in optimal cutting temperature (OCT) media (ThermoFisher, Waltham, MA, USA) and sectioned into 7 µm sections using a Leica CM3050-S cryostat (Leica Biosystems, Wetzler, Germany).

*HSV-2 Antigen IF:* HSV-2 antigens were visualized with immunofluorescent staining using a fluorescein isothiocyanate (FITC) conjugated polyclonal anti-HSV 1/2 antibody (Dako) at a 1:100 dilution. An Olympus IX71 inverted fluorescence microscope was used to visualize slides and images were captured with Olympus cellSense software (Olympus America, Center Valley PA, USA).

*HSV-2 LAT FISH:* Cryosections of DRG from the above animals were probed for the presence of LAT using the RNAscope Multiplex Fluorescent V2 Assay (ACD Bio) following the manufacturer’s protocol. Prior to DAPI nuclear staining and mounting of the coverslip, DRG were immunostained for sensory neuronal marker PGP9.5 to visualize sensory neurons. For IF, slides were washed in 1X RNAScope buffer twice for two minutes each and then rinsed in 1X PBS as described. Slides were blocked at room temperature for 30 min in 1X PBS, 0.1% Triton, and 5% normal donkey serum (NDS). Blocking solution was poured off without rinsing and then a polyclonal IgG rabbit anti-PGP9.5 primary antibody at a 1:250 dilution (Abcam # ab15503) in 1X PBS and 1% NDS was applied. Slides were kept at 4 °C overnight and then rinsed three times in 1X PBS for five minutes each. Slides were re-blocked at room temperature for 30 min in 1X PBS with 3% NDS. A polyclonal IgG donkey-anti-rabbit secondary antibody conjugated to AlexaFluor594 at 1:1000 dilution (Thermo Fisher, # A-21207, Waltham MA, USA) in 1X PBS with 1% NDS was applied and incubated at room temperature for one hour. Slides were washed 3X for 5 min in 1X PBS and DAPI was applied for 30 s as a nuclear stain. Slides were washed in 1X PBS and coverslips were mounted using ProLong Gold Antifade (Thermo Fisher, Waltham MA, USA). Slides were visualized as described above. The number of neurons with latent HSV-2 were counted in four consecutive cryosections and reported as the percentage of latently infected neurons per 500 neurons counted.

### 2.7. Serum Immunoassays

*HSV-2 IgG ELISA:* IgG HSV-2 antibody titers were measured using HSV-2 strain MS virions as previously described [43]. Briefly, purified HSV-2 virions in carbonate buffer (pH = 9.6) were incubated overnight at room temperature in 96-well microtiter plates enclosed in a humidity chamber and allowed to adsorb. Excess antigen was removed by washing the wells three times with PBS-Tween 20. Serial dilutions of serum were added to each well, and the plates were incubated at 37 °C for 2 h. Wells were washed three times with PBS-Tween 20 and then incubated with alkaline-phosphatase-conjugated anti-guinea pig detection antibodies (1:2000 dilution, Southern Biotechnology, Birmingham, AL) for 2 h at room temperature. Wells were washed three times with PBS-Tween 20 and incubated with para-nitrophenyl phosphate substrate for 2 h at room temperature and then overnight at 4 °C covered in aluminum foil. The optical density at 450 nm was measured using a Clariostar microplate reader (BMG Labtech, Ortenberg, Germany) with background correction at the 540 nm wavelength.

*Immunogenicity by ABVIC (antibody-binding to virus-infected cells):* Pan-HSV-2 IgG antibodies were measured using HSV-2 strain MS-infected Vero cells as follows: guinea pig serum was diluted 1:3200 in PBS-S-Ig (50 mL PBS-S, 800 µg Donkey Gamma Globulin, 800 µg Goat Gamma Globulin) and 40 µL of diluted serum was added to a well of 96-well microplate. 160 µL of ABVIC test cells (a mixture of 7.5 × 10^4^ of uninfected (UI) cells and 7.5 × 10^4^ HSV-2 MS carboxyfluorescein succinimidyl ester (CFSE)-labeled infected cells) was added to the well containing diluted serum. The serum samples were incubated with ABVIC test cells for 2 h on microplate shaker (700 rpm, room temperature). The cells were pelleted by centrifugation for 3 min at 1800 rpm. The supernatant was decanted, and each sample well was washed once with 200 µL of PBS-S (500 mL of 1X PBS, 2.0% DES, 0.1% NaAzide). The cells were pelleted using centrifugation for 3 min at 1800 rpm, and supernatant was decanted. Alexa Fluor 647-conjugated AffiniPure Donkey anti-guinea pig IgG (H+L) was diluted 1:1200 in PBS-S-Ig. 100 µL of IgG dilution was added to each well. The samples were incubated for 1 h, on microplate shaker (700 rpm, room temperature). The cells were pelleted using centrifugation, and supernatant was decanted. Three washes were performed with PBS-S, using 150 µL/well for the first wash and 200 µL/well for the second and third washes. The samples were resuspended in 130 µL of PBS-S and analyzed on CytoFLEX flow cytometer (Beckman Coulter, Brea, CA, USA) and mean fluorescent intensity (MFI) was collected for uninfected (UI) cells and HSV-2 cells. Signal-to-noise (S/N) ratios were calculated for each sample by dividing the MFI for HSV-2-infected cells by the MFI for UI cells. Means and standard deviations for the S/N ratios were calculated and graphed.

*Neutralization Assay:* Serum samples were diluted 1:25 in RPMI-1640 containing 10% heat-inactivated fetal bovine serum (Invitrogen, complete medium) in 96-well microtiter plates and serially diluted 2-fold. Guinea pig complement (Rockland Immunochemicals, Gilbertsville PA, USA) was then diluted 1:20 in complete medium containing 5.94 × 10^4^ PFU/mL HSV-2 MS and added to each well (110 µL, total volume). The plates were gently mixed and incubated at 37 °C with 5% CO_2_ for 90 min. Confluent Vero cells were then exposed to the virus-serum dilution for 1 h, decanted, and incubated for 24 h in complete medium. Neutralization titers were reported at which a 50% reduction in cytopathic effect was observed by masked observer.

### 2.8. Statistical Analysis

Statistical analyses were performed in Excel and GraphPad Prism. Results were analyzed by ANOVA and post hoc tests, as described in each figure caption. Error bars are defined in the caption for each figure.

## 3. Results

### 3.1. Clinical Assessment in Guinea Pigs

Guinea pigs are an excellent animal model to assess vaccine or antiviral effects on HSV-2 recurrences, as the virus spontaneously and reliably reactivates to cause recurrent lesions and viral shedding following genital infection. As an initial goal is to assess the vaccine candidates as potential therapeutics to prevent recurrent disease in individuals who are already infected with HSV-2, we sought to assess the safety of the vaccine candidates in the guinea pig model.

Groups of female guinea pigs were inoculated with previously described recombinant viruses HSV-2 0ΔNLS (RVx201) or HSV-2 0ΔRING (RVx202) either vaginally (*n* = 3) or intradermally in the skin of the inner thigh (*n* = 16) [40]. Control groups were also inoculated either vaginally (*n* = 5) or intradermally (*n* = 16) with wild type parent virus HSV-2 strain MS, intradermally with Vero cell lysate (*n* = 3) or intradermally with PBS (*n* = 3). All guinea pigs were monitored daily for acute disease, clinical recurrences, and viral shedding.

**Figure 1:**
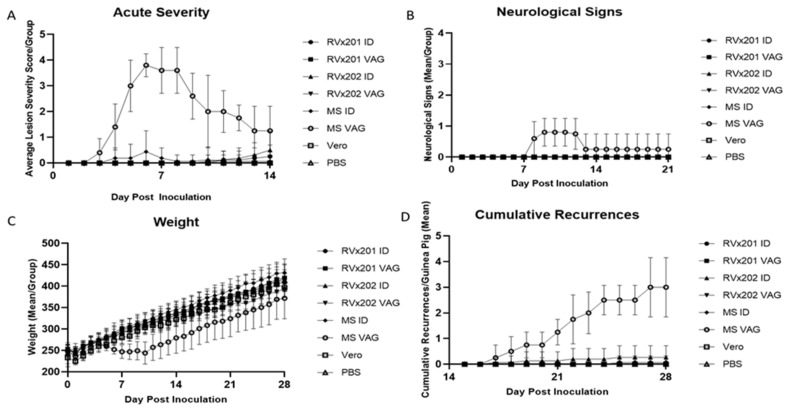
Clinical indicators of disease/distress in inoculated guinea pigs. Following intradermal (ID) or vaginal (VAG) inoculation of guinea pigs with RVx201, RVx202, WT HSV2 MS or ID inoculation with PBS or Vero cell lysate, guinea pigs were monitored for acute disease severity (1–14 dpi), neurological signs (1–21 dpi), weight loss (1–28 dpi), and clinical recurrences (15–28 dpi). (**A**) Acute disease severity was scored on a 4-point severity scale, as follows: 1 = redness/swelling, 2 = 1–2 lesions, 3 = 3–6 lesions, 4 = >6 lesions or a coalescence of lesions. Vaginal infection with WT HSV2 MS produced typical acute disease severity, with significantly greater disease severity compared to all other groups (*p* < 0.0001). ID infection with WT HSV2 MS produced injection site lesions in 5 of 16 animals, beginning 4 dpi. ID infection with RVx201 and RVx202 produced infection site “bumps” or lesions in 7 of 16 and 6 of 16 animals, respectively, starting 10 dpi. (**B**) Guinea pigs were monitored for the development of neurological signs, including urinary retention and/or hindlimb weakness/paralysis. Only VAG infection with WT HSV2 MS produced neurological signs, which resolved in all but one animal by 13 dpi (AUC *p* < 0.0001 compared to all other groups). (**C**) Weight in grams were recorded daily for each guinea pig within each group and reported as average group weight for the duration of the study (28 days). (**D**) Cumulative recurrences were recorded for each guinea pigs, reported as the mean of cumulative recurrences for each group. Vaginal recurrences were defined as vesicular lesions on the external genital epithelium and intradermal recurrences were defined as a vesicular lesion at the site of injection on the inner thigh. GP VAG experienced significantly more recurrences compared to all other groups (*p* < 0.0001). Data are presented as mean ± SD. *p* values were determined by ANOVA and post hoc Tukey’s test.

During the acute period of infection 1–14 days post inoculation (dpi), no significant signs of acute disease were noted in the RVx201, RVx202, Vero cell lysate, or PBS groups inoculated intradermally (ID) beyond mild injection site redness/swelling in some animals (Figure 1A). Mild injection site redness/swelling was noted in 43.75% (7 of 16) of the guinea pigs in the RVx201 ID group beginning 10 dpi, in 37.5% (6 of 16) of the guinea pigs in the RVx202 ID group beginning 10 dpi, and 31.25% (5 of 16) of guinea pigs in the WT HSV-2 MS group beginning 3 dpi. Vaginal infection with wild type HSV-2 MS produced typical acute lesions, with a peak severity score of 3.8 (on a 4-point scale) 6 dpi (*p* < 0.0001 compared to all other groups). In contrast, guinea pigs infected vaginally with RVx201 or RVx202 developed no genital lesions (Figure 1A).

Neurological signs, including urinary retention and/or hindlimb weakness/paralysis was noted in 80% of (4 of 5) of the guinea pigs in the WT HSV-2 MS VAG group beginning 8 dpi (Figure 1B, *p* < 0.0001 compared to all other groups). No such neurological signs were observed in the RVx201, RVx202, Vero cell lysate, or PBS groups inoculated ID or VAG.

No significant weight loss was noted in guinea pigs inoculated with RVx201 or RVx202 during the study period, regardless of route of inoculation (Figure 1C). In contrast, guinea pigs inoculated vaginally with wild type HSV-2 MS lost weight during the acute period of infection, from 5–10 dpi (Figure 1C), correlating with the peak of acute infection (*p* < 0.05 compared to all other groups 8–11 dpi).

Following acute infection, guinea pigs were observed daily for recurrences of genital or injection site lesions (15–28 dpi). Only animals vaginally inoculated with WT HSV-2 MS developed recurrent genital lesions, defined as vesicular lesions on the external genitals (Figure 1D, *p* < 0.0001 compared to all other groups). Animals inoculated vaginally with RVx201 or RVx202 did not develop genital recurrences.

**Figure 2:**
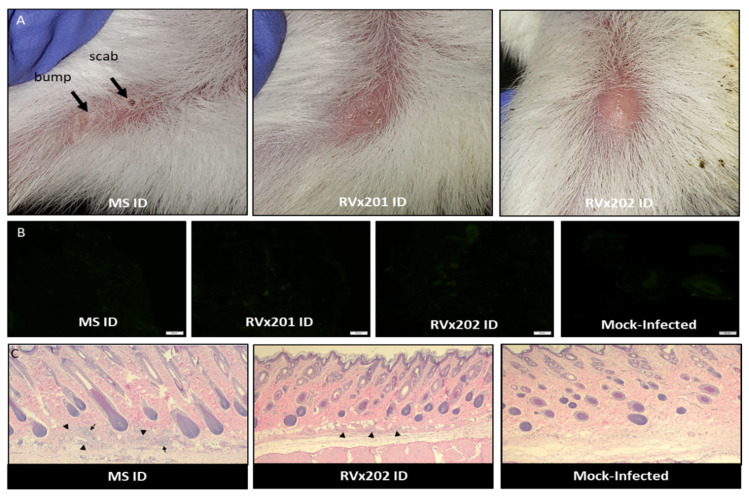
Pathology of lesions at intradermal injection site. (**A**) Representative images of lesions at the injection site following intradermal inoculation with HSV-2 MS (GP48) RVx201 (GP14) and RVx202 (GP27). Guinea pig inoculated with HSV-2 MS developed initial injection site vesicular lesion 6 dpi, which by 10 dpi had progressed to a scab by 10 dpi and a second “bump” appeared. (**B**) Representative images of injection site skin from guinea pigs inoculated ID with HSV-2 MS, RVx201, RVx202, and mock-infected stained for the presence of HSV-2 antigens with a polyclonal antibody. No viral antigen was present in any of the examined injection site skin samples. (**C**) Histopathology examination of injection site skin demonstrated small clusters of leukocytes (arrowheads), with minimal increase in mature collagenous stroma (fibrosis) associated with minimal chronic inflammation at the dermal/subcutis border in animals inoculated with HSV-2 MS and RVx202 (RVx201 was similar to RVx202). Mock-infected guinea pigs injected with PBS or Vero cell lysate showed no such changes.

Among animals inoculated intradermally, one guinea pig inoculated with RVx201 developed a single lesion 25 dpi at the injection site on the inner thigh, appearing as a vesicular lesion and lasting for 3 days; four guinea pigs inoculated with RVx202 developed similar injection site lesions, each experiencing a single vesicular lesion at the injection site. In addition, two additional animals inoculated with RVx201 and four additional animals inoculated with RVx202 developed “bumps” at the injection site during the recurrence period of observation (15–28 dpi) (Figure 2A). Injection sites that were associated with either vesicular skin lesions or “bumps” did not have detectable viral antigen when assessed using immunofluorescence (IF) with a polyclonal HSV-2 antibody (Figure 2B). Pathological analysis of injection site lesions/bumps by Histo-Scientific Research Laboratories (Mount Jackson, VA, USA) reported that the injection site was noted to have small clusters of leukocytes at the subcutis border, with minimal increase in mature collagenous stroma (fibrosis) associated with minimal chronic inflammation (Figure 2C).

### 3.2. Low Level Transient Viral Replication Occurred after Vaginal Inoculation with RVx202 but Not with RVx201 or with Intradermal Inoculation

**Figure 3:**
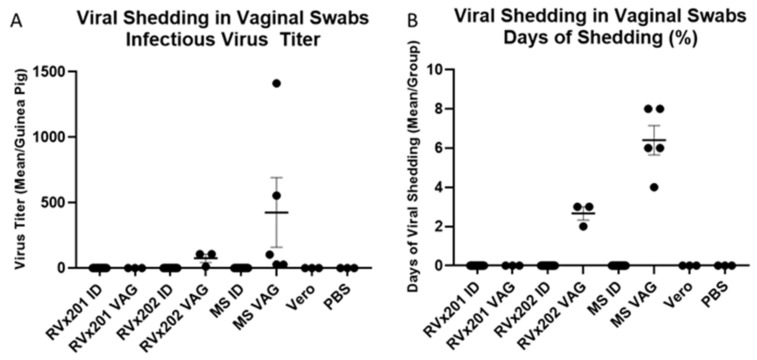
Guinea pigs were swabbed vaginally daily for the assessment of viral shedding. (**A**) Viral titers in vaginal secretions were determined via plaque assay. Guinea pigs vaginally infected with WT HSV-2 MS and RVx202 shed infectious virus in vaginal secretions. (**B**) The percentage of days that virus was shed in vaginal secretions was also determined. Guinea pigs vaginally infected with WT HSV-2 MS and RVx202 shed infectious virus in vaginal secretions. Guinea pigs infected ID with RVx201, RVx202, or MS shed significantly less infectious virus (*p* < 0.0001) for significantly fewer days (*p* < 0.0001) compared to MS VAG infected animals. Data are presented as mean ± SEM. P values were determined by ANOVA and post hoc Tukey’s test.

Guinea pigs were swabbed vaginally daily to assess shedding of HSV-2 in vaginal secretions. Shedding was not detected in vaginal secretions of guinea pigs inoculated ID with RVx201, RVx202, HSV-2 MS, Vero cell lysate, or PBS. However, virus was detected in vaginal swabs collected from all three guinea pigs vaginally inoculated with RVx202 (5–2600 infectious virus particles on individual days, averaging 12.0–106.8 pfu over the 28 days of observation) from 1–3 dpi and from all five guinea pigs vaginally inoculated with WT HSV-2 MS (2.5–39,500 infectious virus particles on individual days, averaging 27.4–1411.7 pfu over the 28 days of observation) from 1–12 dpi (Figure 3A). Guinea pigs infected VAG with RVx202 shed infectious virus for an average of 9.5% of days (7.1–10.7% of days, 2–3 of the 28 days of observation) while those infected with WT HSV-2 MS shed for an average of 22.9% of days (14.3–28.6% of days, 4–8 of the 28 days of observation) (Figure 3B). Guinea pigs infected with RVx201 did not shed infectious virus at any time during the study period. Thus, guinea pigs infected ID with RVx201 or RVx202 shed significantly less infectious virus (*p* < 0.0001) and for significantly fewer days (*p* < 0.0001) compared to guinea pigs infected VAG with WT HSV-2 MS.

### 3.3. Neither Vaccine Candidate Established Latency or Detectable Replicating Virus in Lumbosacral Dorsal Root Ganglia

**Figure 4:**
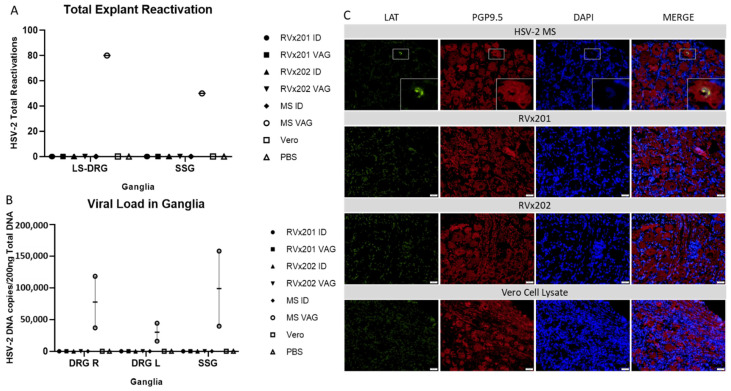
Establishment of latency in lumbosacral dorsal root ganglia (DRG) and sacral sympathetic ganglia (SSG). Following euthanasia, sensory and autonomic ganglia including LS-DRG, SSG, TG, SCG, and brain were assessed for HSV latency (TG, SCG, brain not shown, all negative). (**A**) Tissues were maintained in culture for 7 days and assessed daily for infectious virus released into media by plaque assay. Only guinea pigs inoculated VAG with MS reactivated. (**B**) DNA was extracted and HSV-2 quantified via qPCR targeting HSV-2 thymidine kinase (LOD: 10^2^ copies) in right DRG (R-DRG, *p* = 0.0071 vs. PBS), left DRG (DRG-L, *p* = 0.0030 vs. PBS), and SSG (*p* = 0.0174 vs. PBS). (**C**) Fluorescent in situ hybridization (FISH) for latency associated transcript (LAT) in LS-DRG. Cryosections of LS-DRGs were stained for LAT using FISH (green) and for sensory neuronal marker PGP9.5 using immunofluorescence (red), and counterstained using DAPI (blue). The individual color channels were then merged (far right). LAT was detected by FISH only in LS-DRG of guinea pigs infected with WT HSV2 MS, not in those infected with RVx201, RVx202, or mock-infected with Vero cell lysate or PBS. Data are presented as mean ± SEM. P values were determined by ANOVA and post hoc Tukey’s test.

To assess the ability of RVx201 and RVx202 to establish latency in ganglia innervating the site of inoculation, several methods were employed. Initially, an explant reactivation method was used to assay for infectious virus. In this case, infectious virus was not recovered from lumbosacral dorsal root ganglia (LS-DRG), sacral sympathetic ganglia (SSG), superior cervical ganglia (SCG), trigeminal ganglia (TG), or cerebral homogenate as demonstrated by the failure to produce visible plaques over the seven-day observation period, except DRG and SSG of the guinea pig VAG inoculated with WT HSV-2 MS (Figure 4A). Second, we quantified viral DNA in homogenates of ganglia and brain. Although viral DNA was detected in both left and right DRG and SSG of guinea pigs VAG inoculated with WT HSV-2 MS, using qPCR targeting HSV-2 thymidine kinase (TK) gene, no viral DNA was detected in any ganglia in either ID or VAG infected guinea pigs receiving RVx201, RVx202, Vero cell lysate or PBS (limit of detection: 10^2^ copies) (Figure 4B). No viral DNA was detected in TG, SCG or brain homogenates of any guinea pigs (not shown). Third, ganglia were assessed for expression of viral antigens and latency associated transcript (LAT). Viral antigen was not detected by immunofluorescence (IF) in any ganglia from any animals in the study, indicating that active viral replication was not occurring at study end 28 dpi. Combined fluorescent in situ (FISH) hybridization-IF targeting LAT identified expression in 3.38% of DRG neurons (17 neurons positive of 500 neurons counted across multiple sections) from guinea pigs inoculated with WT HSV-2 MS (n = 1 VAG, 0 ID) but no LAT positive neurons were detected in ganglia of guinea pigs inoculated with RVx201, RVx202, Vero cell lysate or PBS (Figure 4C).

### 3.4. Vaccine Candidates Induce Serum Antibody Response Following Intradermal Inoculation

**Figure 5:**
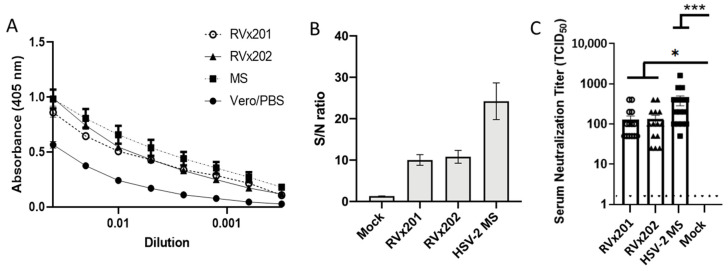
Serum antibody response. Guinea pigs (*n* = 3–15/group) were inoculated ID with RVx201, RVx202, MS, or control (Vero cell lysates or PBS) intradermally. Twenty-eight days post infection serum was collected and assayed for reactivity to HSV-2 antigen by (**A**) ELISA or (**B**) ADVIC test. Average sample-to-noise (S/N) ratios represent pan-HSV2 immune response. (**C**) Serum neutralization titers against HSV-2. Data are presented as mean ± SEM. *** *p* < 0.001, * *p* < 0.05 comparing the indicated groups by Kruskal–Wallis non-parametric ANOVA and Dunn’s multiple comparison test.

To assess the development of antibodies in response to inoculation with RVx201 and RVx202, sera collected from the guinea pigs were analyzed by ELISA and antibody-binding to virus-infected cells (ABVIC) assays to quantify HSV-2 IgG levels. Guinea pigs inoculated intradermally with RVx201 and RVx202 developed anti-HSV-2 antibodies, as shown by ELISA (Figure 5A) and by ABVIC (Figure 5B). To assess the neutralizing capabilities of the serum antibodies, plaque reduction neutralization assays were performed, demonstrating that antibodies induced in response to intradermal inoculation of RVx201 and RVx202 were able to effectively neutralize HSV-2 (Figure 5C).

## 4. Discussion

In this study, we assessed the safety of two previously described HSV-2 recombinant viruses with targeted mutations in ICP0, HSV-2 0ΔNLS (designated RVx201) and HSV-2 0ΔRING (designated RVx202), as potential vaccine candidates by intradermal inoculation in a guinea pig model. Our findings support further investigation of RVx201 as a live attenuated vaccine candidate for HSV-2.

While the creation of an effective HSV-2 vaccine has proven elusive, hope is offered by the existence of vaccines against other persistent viruses, including alphaherpesvirus varicella zoster virus (VZV) and other viruses that cause genital infections (e.g., human papillomavirus, HPV). The existence of a live-attenuated herpesviruses vaccine against VZV and its dual applications as a preventative vaccine against primary disease in the young (e.g., varicella or chickenpox) and as a therapeutic against recurrent disease in those already infected (e.g., herpes zoster or shingles) demonstrates not only the potential safety of live-attenuated herpesvirus vaccines but also the potential for an HSV-2 vaccine to have both preventative and therapeutic applications [44,45]. Although most vaccines are delivered by the subcutaneous or intramuscular routes, intradermal delivery would be expected to induce a superior response against HSV due to the abundance of antigen-presenting cells in the dermis. Previous studies have shown that intradermal delivery with reduced doses of influenza and vaccinia vaccines produces a response equivalent to standard doses delivered by the conventional routes [46,47,48]. Equivalent immune responses and protection against vaccinia-virus challenge were induced with intradermal doses of modified vaccinia Ankara ten-fold lower than those delivered by intramuscular or subcutaneous injection [46]. Both vaccine candidates are intended to be administered via an ID injection, which may suggest limitations for the development of protective mucosal immunity; however, results from the HPV vaccine show these concerns may be unsubstantiated. The HPV vaccine, a virus-like particle composed of the major capsid protein L1 of HPV, is administered as an intramuscular injection and has been shown to produce protective mucosal immunity engaging both humoral and cell-mediated immune components against HPV at multiple portals of entry [49,50]. Only one HSV-2 vaccine candidate had been tested in clinical trials using an ID injection prior to the development of RVx201 and RVx202 (Table 1). This DNA plasmid-based therapeutic vaccine candidate targeting glycoprotein D of HSV-2 (gD2) elicited a gD2-specific cell-mediated response in ≈40% of patients and a gD2-specific antibody response in ≈50% of patients. Viral shedding in genital swabs was reduced by 50% compared to placebo only after a booster following a prime, boost, boost vaccination series [51,52]. These results demonstrate the ability of HSV-2 vaccine candidates delivered via an ID injection to elicit cellular and adaptive immune responses, as well as achieve therapeutic endpoints. The results, however, are less than robust and are not present in the majority of those vaccinated, which may be a result of using a plasmid as the vaccine platform. These shortcomings may be resolved by the delivery of live-attenuated HSV-2 vaccine candidates such RVx201 and RVx202, which represents a truly novel approach in HSV-2 vaccinology as no such vaccine platform has been paired with ID injection.

**Table 1:** A brief review of HSV-2 vaccine candidates having completed various clinical trial phases from 1990–2020. Clinical trials include those registered with clinicaltrials.gov as well as relevant unregistered trials from academic universities and agencies outside the United States who collaborated with US institutions. gD2: glycoprotein D, gB2: glycoprotein B, ICP: infected cell protein, UL: unique long.

Vaccine Type	Agency	Antigen	Trial Phases Completed	Efficacy of HSV-2 Infection Prevention or Disease Amelioration
Subunit (monovalent)	GlaxoSmithKline	gD2	1–3 [53,54,55,56,57,58,59,60,61]	Infection: Not effective at preventing HSV-2 mediated infection/disease. Mild efficacy at preventing HSV-1 mediated infection/disease but not in men or HSV-1+ women [62,63,64].
Subunit (bivalent)	Genocea Biosciences	gD2, ICP4 [65]	1–2 [66,67,68,69,70,71]	Disease: Transient six-month reduction in viral shedding. Reduced recurrence rates and duration in 20% up to 12 months [72].
	Immune Design	gD2, UL19, UL25 [73]	1–2 (active) [74]	Active study
	Chiron (Novartis)	gD2, gB2	1–2	Infection: 9% reduction over 18 months (Transient 50% reduction during first 5 months vs. placebo) Disease: Frequency of recurrence not reduced [75,76].
Subunit (polyvalent)	Agenus	22 HSV-2 Proteins [77]	1–2 [78,79]	CD4+ and CD8+ T cell responses detected [80]. Phase 2 results not published.
DNA (plasmid)	PowderMed (Pfizer)	ICP0, ICP4, ICP22, ICP27	1 [81,82]	Not published
	Vical	gD2, Tegument [83,84]	1–2 [85,86]	Not published
	Admedus	gD2 [87]	1–2	Disease: Reduced viral shedding ≈50% after booster compared to placebo [51,52].
Replication deficient virus	Sanofi Pasteur	HSV-2 [88]	1 2 (active) [74,89,90]	Active study. Shown to be immunogenic in HSV-1/HSV-2 seronegative participants [91]

To date, the only HSV vaccine to be tested in a Phase III clinical trial was a subunit vaccine based on a recombinant gD2 adjuvanted with alum and lipid A. The vaccine failed to prevent HSV-2 infection although it did induce an anti-gD2 antibody response that waned within six months. The vaccine trial did show limited efficacy at preventing genital infection with HSV-1 as well as limited efficacy at reducing symptoms of HSV-1 infection, which was correlated with participants who had higher anti-HSV gD antibody levels [62]. Another subunit vaccine using both recombinant gD2 and gB2 with a different adjuvant (MF59) demonstrated minimal efficacy at preventing infection between HSV-2 discordant partners (13%) and participants attending a sexually transmitted disease clinic (<5%) over 18 months of observation (Table 1) [75,76]. These results may indicate that other targets beyond glycoprotein D, glycoprotein B, other vaccine platforms beyond subunit vaccines and/or other routes of administration are needed in the development of an anti-HSV-2 vaccine [92].

The previous 30 years of HSV-2 vaccinology (Table 1) has been dominated mostly by the development and clinical testing of recombinant monovalent and bivalent subunit vaccines primarily targeting either gD2 or gB2 alone or in combination [62,63,64,72,75,76]. A singular polyvalent recombinant subunit vaccine was developed, shown to elicit CD4+ and CD8+ T cell responses during a Phase I trial but has yet to report results from subsequent trials [79,80]. Beyond subunit vaccines, DNA plasmid-based vaccines expressing various infected cell proteins (ICPs) or gD2 have also been developed [51,52,81,82,85,86]. None of these previous therapeutic vaccine candidates have consistently reduced viral shedding, frequency of recurrence, and duration of recurrence long-term in the majority of those vaccinated and no preventative vaccine candidates have consistently reduced rates of infection for all vaccinees regardless of sex or HSV-1 serostatus. Given this history, the development and testing of historically underdeveloped vaccine platforms (e.g., live-attenuated replication deficient HSV-2) presents an attractive alternative to subunit and DNA plasmid-based HSV-2 vaccines.

Several live-attenuated HSV-2 vaccine candidates targeting various components of HSV-2 (e.g., ICP10, gE2, and UL24) have been developed pre-clinically and tested in animal models but none have completed human trials [93,94,95,96,97,98]. A replication-competent HSV-2 gE2 deletion mutant (gE2-del virus) deficient in epithelial cell-to-neuron spread was investigated as a therapeutic vaccine candidate in guinea pigs previously infected with HSV-2. Immunization resulted in a reduction in recurrent vaginal lesions, infectious viral shedding, and latently infected DRGs following inoculation using a prime boost regimen [97]. In a concomitant challenge study in guinea pigs, 50% of HSV-2 gE2-del inoculated animals (5 of 10) were protected from developing disease following intravaginal challenge with WT HSV-2. Of the animals that did develop recurrent disease (1 of 10), the rate of recurrence and titer of infectious virus shed was reduced [97]. While these results demonstrating amelioration of recurrent disease were promising despite a small sample size, additional development of this vaccine candidate has not been published since 2012.

An attenuated HSV-2 G strain mutant deficient for the protein kinase domain of the large ribonucleotide reductase subunit ICP10 (ICP-10ΔPK) was found to prophylactically protect 90% of vaccinated Hartley guinea pigs from primary genital disease following a prime, boost regime and reduced the severity of disease in those remaining. In a separate experiment, prophylactic vaccination following a prime, boost, boost regime reduced the proportion of DRGs containing HSV-2 DNA (32% vs. 70%) as well as the overall concentration of HSV-2 DNA (99.4% reduction) post challenge compared to controls, indicating protection from the establishment of latency. Of note, latency was not established by the attenuated virus. When tested as a therapeutic vaccine using a prime, boost regimen, the attenuated virus reduced the number of recurrences of vaginal lesions as well as the duration of recurrence following intravaginal infection with WT HSV-2 [93,94]. The immune response elicited by ICP-10ΔPK was predominantly a Th1 response as evident by the presence of an increased concentration of HSV-2 specific CD4+ IFN γ secreting cells, increased dendritic cell IL-12 secretion, and the presence of IgG_2_a in greater concentrations than IgG_1_. Additionally, present was a robust HSV-2 specific CD8+ T cell response that when adoptively transferred from ICP-10ΔPK immunized mice to non-ICP-10ΔPK mice prevented infection after challenge with WT HSV-2 [95]. These results suggest that attenuated HSV-2 vaccine candidates may be able to generate a more Th1 biased response targeting destruction of HSV-2 infected cells rather than the more Th2 biased response generated by WT HSV-2 infection, which has been indicated as a factor limiting the efficacious destruction of HSV-2-infected cells. A small scale (*n* = 32) Phase I/II clinical trial of the ICP-10ΔPK mutant was conducted in public hospitals in Mexico City, Mexico in patients 18–55 years of age with recurrent HSV-2 disease characterized by ≥5 lesions/year. ICP-10ΔPK was tested as a therapeutic vaccine following SQ injection 7, 17, and 28 days after the occurrence of a lesion. The vaccine candidate was well tolerated with minimal side effects (e.g., headache, injection site redness, myalgia) and recurrent disease was not observed in 37.5% (*n* = 9) of the ICP-10ΔPK recipients during the 180 days follow up period compared to 100% in the placebo group. Of those who had recurrent disease, the number of recurrences, duration of recurrence, and associated symptoms of recurrence (e.g., vesicles, pain, itching) were all reduced [96]. Results from studies of this vaccine candidate demonstrates that safety and therapeutic endpoints can be achieved using a live-attenuated HSV-2 vaccine candidate, however no additional development of this vaccine candidate has been published since 2002.

A Th1 skewed response, like that observed with ICP-10ΔPK, was also observed in mice immunized with a UL24-βgluc insertion mutant (HSV-2 186 parental strain). Lymphocytes isolated from the spleens of mice inoculated with UL24-βgluc were shown to be cytolytic to HSV-2 infected cells and to express elevated IFN-γ upon stimulation. Additionally, the humoral response was like that observed previously with greater concentrations of IgG_2_a than IgG_1_. Mice inoculated with UL24-βgluc were subsequently protected from lethal disease following intravaginal challenge with WT HSV-2. In guinea pigs immunized with UL24-βgluc following a prime boost regimen prior to intravaginal challenge with WT HSV-2, the mean number of lesions as well as the cumulative number of recurrences was significantly reduced compared to sham-inoculated animals. Of note, HSV-2 DNA could not be detected in the DRGs of 75% of the guinea pigs vaccinated with UL24-βgluc prior to challenge with WT virus [98]. These results are promising and suggest that a Th1 skewed response may be commonly generated, recurrent disease symptoms can be ameliorated, and that establishment of latency can be interrupted following inoculation with live attenuated HSV-2 vaccine candidates. However, additional development of this vaccine candidate has not been published since 2014.RVx201 was used in a previous animal study as a polyvalent immunogen to identify correlates of immunity of protection from HSV-2, since most such studies have relied on monovalent or bivalent vaccines, which do not represent the full HSV-2 proteome. RVx201 was found to reduce viral shedding in vaginal swabs after intravaginal challenge with WT HSV-2 following prime boost immunizations in the animal’s footpads (e.g., mice and guinea pigs). Concentrations of HSV-2 pan-IgG in pre-challenge sera of both animal types was linearly correlated with this reduction in vaginal shedding compared to naïve animals. These results are in line with previously observed results discussed above and suggested the need for further study of RVx201 as a potential HSV-2 vaccine candidate using immunization via a more clinically relevant site/method (e.g., intradermal) and with assessment of overall safety (e.g., tolerability and ability of the vaccine candidate to establish latency at the injection site and clinically relevant neuronal tissues) [99].

Intradermal inoculation of RVx201 and RVx202 does not cause significant disease or distress beyond mild swelling and redness at the injection site related to minor inflammation of the subcutis. Neither vaccine candidate produces recurrent disease or lesions at the injection site as demonstrated by the lack of detection of viral antigens at the injection site. Additionally, these vaccine candidates do not appear to establish latency in sensory or autonomic ganglia, including LS-DRG, SSG, SCG, or TG, following ID or VAG inoculation, as demonstrated by the lack of detection of viral DNA via qPCR, LAT via RT-qPCR or FISH, viral antigen via IF, or infectious virus via explant reactivation. No infectious virus was shed in vaginal secretions following ID inoculation with either RVx201 or RVx202, the proposed main administration method of the vaccine candidates in humans. A single intradermal inoculation of either RVx201 or RVx202 was sufficient to elicit an anti-HSV-2 IgG response with neutralizing antibody titers as demonstrated by ELISA, ABVIC, and neutralization assay.

## 5. Conclusions 

In summary, RVx201 appears to be safe in guinea pigs, as it did not produce observable signs of disease, did not establish detectable latency, and did not produce recurrences. RVx202 produced minor injection site inflammation, viral shedding in vaginal secretions at early time points following vaginal inoculation (but not following intradermal inoculation) but did not establish detectable latency or produce recurrences. As the findings suggest these two novel live-attenuated vaccine candidates are safe, their efficacy at preventing infection and/or reducing recurrent disease will be investigated.

## Data Availability

The data that support the findings of this study are available but restrictions apply to the availability of these data, which were used under license for the current study, and so are not publicly available. Data are however available from the authors upon reasonable request and with permission of Rational Vaccines Inc.

## Institutional Animal Care and Use Committee Statement

The study was conducted according to the guidelines of the NIH and Office of Laboratory Animal Welfare, with approval from the Virginia Tech Institutional Animal Care and Use Committee, IACUC #18-155 8/6/2018.
